# Cathepsin B increases ENaC activity leading to hypertension early in nephrotic syndrome

**DOI:** 10.1111/jcmm.14387

**Published:** 2019-07-31

**Authors:** Alexey Larionov, Eileen Dahlke, Madlen Kunke, Luis Zanon Rodriguez, Ina M. Schiessl, Jean‐Luc Magnin, Ursula Kern, Abdel A. Alli, Geraldine Mollet, Oliver Schilling, Hayo Castrop, Franziska Theilig

**Affiliations:** ^1^ Institute of Anatomy, Department of Medicine University of Fribourg Fribourg Switzerland; ^2^ Institute of Anatomy Christian Albrechts‐University Kiel Kiel Germany; ^3^ Institute of Physiology University of Regensburg Regensburg Germany; ^4^ Service LaboratoireHôpital Fribourg Fribourg Switzerland; ^5^ Institute of Molecular Medicine and Cell Research, Faculty of Medicine University of Freiburg Freiburg Germany; ^6^ Department of Physiology and Functional Genomics University of Florida Gainesville Florida; ^7^ Laboratory of Hereditary Kidney Diseases, INSERM UMR 1163 Université Paris Descartes‐Sorbonne Paris Cité, Imagine Institute Paris France; ^8^ BIOSS Center for Biological Signaling Studies University of Freiburg Freiburg Germany

**Keywords:** epithelial sodium channel, focal segmental glomerulosclerosis, hypertension, nephrotic syndrome

## Abstract

The *NPHS2* gene, encoding the slit diaphragm protein podocin, accounts for genetic and sporadic forms of nephrotic syndrome (NS). Patients with NS often present symptoms of volume retention, such as oedema formation or hypertension. The primary dysregulation in sodium handling involves an inappropriate activation of the epithelial sodium channel, ENaC. Plasma proteases in a proteinuria‐dependent fashion have been made responsible; however, referring to the timeline of symptoms occurring and underlying mechanisms, contradictory results have been published. Characterizing the mouse model of podocyte inactivation of *NPHS2* (Nphs2^∆pod^) with respect to volume handling and proteinuria revealed that sodium retention, hypertension and gross proteinuria appeared sequentially in a chronological order. Detailed analysis of Nphs2^∆pod^ during early sodium retention, revealed increased expression of full‐length ENaC subunits and αENaC cleavage product with concomitant increase in ENaC activity as tested by amiloride application, and augmented collecting duct Na^+^/K^+^‐ATPase expression. Urinary proteolytic activity was increased and several proteases were identified by mass spectrometry including cathepsin B, which was found to process αENaC. Renal expression levels of precursor and active cathepsin B were increased and could be localized to glomeruli and intercalated cells. Inhibition of cathepsin B prevented hypertension. With the appearance of gross proteinuria, plasmin occurs in the urine and additional cleavage of γENaC is encountered. In conclusion, characterizing the volume handling of Nphs2^∆pod^ revealed early sodium retention occurring independent to aberrantly filtered plasma proteases. As an underlying mechanism cathepsin B induced αENaC processing leading to augmented channel activity and hypertension was identified.

## 1 INTRODUCTION

The *NPHS2* gene, encoding the slit diaphragm protein podocin, accounts for 43% of familial and 10% of sporadic forms of nephrotic syndrome (NS).^1^, ^2^ Conditional inactivation of podocin in adult mice is a novel model system for NS resulting from focal segmental glomerulosclerosis (FSGS),^3^ which recapitulates human disease formation.

In the NS, the underlying dysregulation in volume homeostasis was shown to be an intrarenal defect^4^ located beyond the distal convolutions in the renal connecting tubule and collecting ducts. Abnormal high activity of the epithelial sodium channel (ENaC) was proven to be the reason for the increased transepithelial sodium reabsorption.^5^ ENaC plays a key role in regulating extracellular fluid homeostasis and blood pressure. Numerous studies of animal models with proteinuria and sodium retention demonstrated increased full‐length subunit expression of ENaC and proteolytical processing of the ENaC subunits alpha and gamma.^6^, ^7^, ^8^, ^9^ In animal models with NS, the increased expression level of ENaC was demonstrated to be independent of its hormonal stimulation. Various attempts in blocking hormones known to activate ENaC did not abolish volume retention.^7^, ^10^ Augmented ENaC activity also results from proteolytic processing of the large extracellular domain of α‐ and γENaC. A dual cleavage event in either subunit releases small intrinsic inhibitory tracts transitioning channels to a more active state.^11^ While furin, an endogenous protease, was shown to cleave αENaC twice, it cleaves the γENaC only once. Additional proteases, including extracellular proteases, are needed for the second incision in γENaC to release the inhibitory tract. Several proteases processing γENaC were identified^12^, ^13^ including plasmin in the development of NS.^14^, ^15^

Regarding the timeline of the appearance of sodium retention and proteinuria, contradictory results have been published. In the rat model of PAN‐induced nephrosis, sodium retention was shown to start before or at the same time as the onset of proteinuria.^7^, ^16^ Consequently, the question arises whether glomerular plasmin leakage is the only mechanism for ENaC‐induced sodium retention. Both, the rat model of PAN‐induced nephrosis and the mouse model of doxorubicin‐induced NS^17^ develop volume retention and oedema very fast within a couple of days, additionally both models show a high number of non‐responders and animal drop‐out during the experiment rendering timeline analysis difficult. The inducible mouse model of podocyte inactivation of *NPHS2* was presented earlier to develop NS with albuminuria, hypercholesteremia and hypertension with progressive podocin loss and at 4 weeks after induction of *NPHS2* deletion, an FSGS is fully established.^3^ Thus, the aim of the study was to characterize this inducible mouse model of podocyte inactivation of *NPHS2* with respect to volume handling and proteinuria, to carefully examine the timeline of the symptom appearance and to identify new mechanism for the dysregulated sodium handling during the development of NS.

We used *Nphs2^fl/fl^*(control) and Nphs2^fl/fl^ *crossbred with* inducible podocyte‐specific *Cre recombinase* transgenic mice, termed Nphs2^∆pod^ hereafter and found that sodium retention and hypertension established before the onset of an unselective gross proteinuria. Increased ENaC channel activity, proteolytic processing of αENaC together with the appearance of proteases in the urine were encountered. Among several lysosomal enzymes identified by proteomic analysis, only cathepsin B was able to cleave αENaC and augment channel activity. Inhibition of cathepsin B influenced the development of hypertension demonstrating its important role in this disease model.

## 2 METHODS

Detailed methods are presented in the supplement files.

### 2.1 Animals and treatments

All animal experiments were conducted according to the NIH Guide for the care and use of Laboratory animals, as well as the Swiss and German law for the welfare of animals and were approved by local authorities (2013_06E_FR, 23614; 2016_28_FR, 28328; V242‐20597/2018). Mice were housed in a SPF facility with free access to chow and tap water and a 12‐hour day/night cycle. Breeding and genotyping was performed as described.^3^ *Nphs2^fl/fl^*(control) and Nphs2^fl/fl^ *crossbred with* inducible podocyte‐specific *Cre recombinase* transgenic mice, termed Nphs2^∆pod^,^3^ were used. For the induction of knockout leading to focal segmental glomerulosclerosis, 6 weeks old Nphs2^∆pod^ and control mice received tamoxifen (33 mg/kg per d for 5 d; Sigma, Buchs, Schweiz) by daily *ip* injection for 4 days in the evening after spot urine sampling and blood pressure measurements. A second set of animal experiments was performed (V242‐20597/2018) using amiloride (Sigma‐Aldrich) 10 µg/g body weight administered i.p. once daily starting on the first day of the experiment, simultaneously with the tamoxifen injection, till the end of the experiment at 14 days. A third set of animal experiments (2016_28_FR, 28328) was conducted using the cathepsin B inhibitor CA‐074Me (MerckMillipore, Darmstadt, Germany). Six weeks old Nphs2^∆pod^ and control received Me‐074 or vehicle by osmotic mini pump (1002, Alzet, USA) for 14 days. Implantation of pumps was performed 1 day after the first tamoxifen injection. CA‐074Me was administered by mini‐pump infusion at a rate of 2.5 mL/h (1 mg/mL in saline with 1.5% DMSO, representing a dose of 0.15 mg/kg/day). Vehicle control infusion was conducted with 1.5% DMSO in saline. All mice entered also completed the experiment.

### 2.2 Presentation of data and statistical analysis

Quantitative data are presented as means ± SEM. Statistical comparisons were performed with the GraphPad Prism Software Package 6 (GraphPad Software Inc, La Jolla, CA, USA). For statistical comparison, the non‐parametric Mann–Whitney *U* test, the parametric two‐tailed Student's *t* test and, where appropriate one‐way ANOVA followed by Tukey, Dunnett or Newman‐Keuls post hoc test was employed. *P* values of less than 0.05 were considered statistically significant.

## 3 RESULTS

### 3.1 Podocin loss leads to nephrotic syndrome

**Figure 1:**
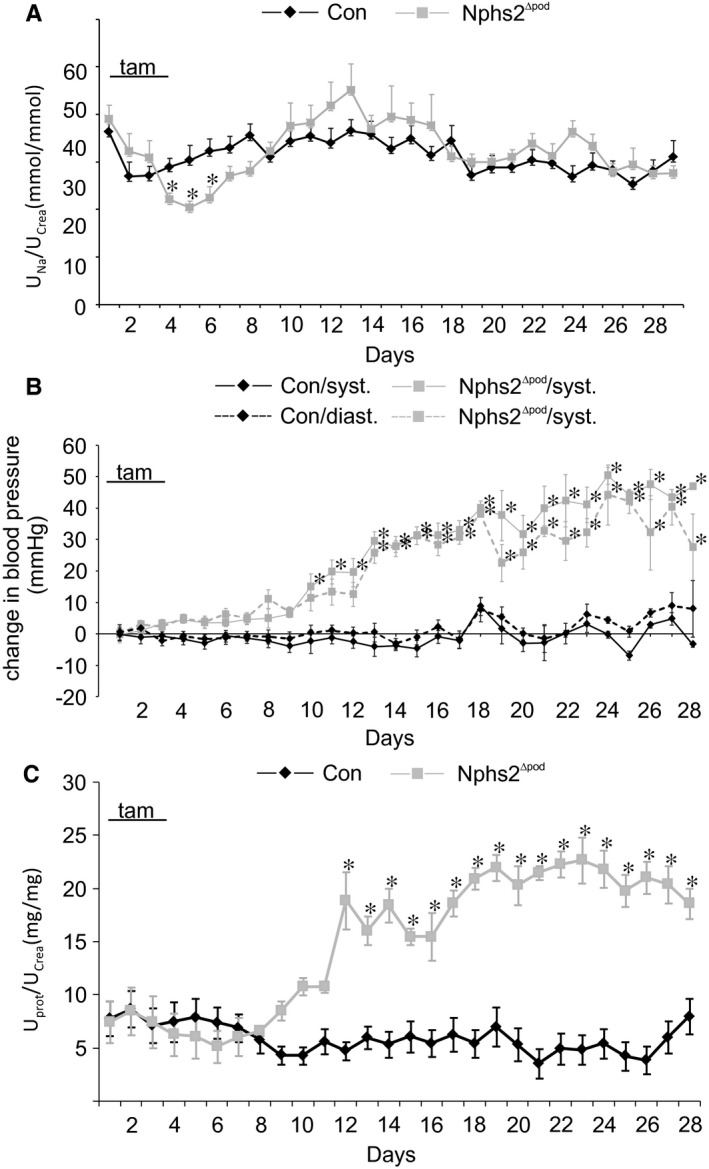
Course of urinary sodium/creatinine ratio, blood pressure and urinary protein/creatinine ratio from Con and Nphs2^∆pod^. A‐C, Daily urinary sodium/creatinine ratio (A), blood pressure (B) and urinary protein/creatinine ratio (C) of control (Con) and Nphs2^∆pod^ during 28 days. Results are arithmetic means ± SEM of n = 5‐7 per group; *P < 0.05. tam indicates the days of tamoxifen injection to induce Nphs2 knockout

Inactivation of podocin resulted in transiently reduced urinary sodium/creatinine ratio, which was significantly reduced between day 4 to 7 after tamoxifen administration (Figure 1A). Systolic and diastolic blood pressure began to rise significantly at day 10 and day 12, respectively, after tamoxifen administration (Figure 1B) and urinary protein/creatinine ratio augmented significantly at day 12 after tamoxifen administration (Figure 1C). Blood pressure and protein/creatinine ratio continued to increase until the end of the experiment at 28 days. Oedema was regularly encountered in all Nphs2^∆pod^ at 3 weeks of the experiment. These results show in detail renal volume handling and proteinuria during the development of NS in Nphs2^∆pod^ and confirm previous published data.^3^, ^18^

### 3.2 Analysis of renal function, morphology and expression of ENaC and its cleavage products in nephrotic syndrome during sodium retention

**Figure 2:**
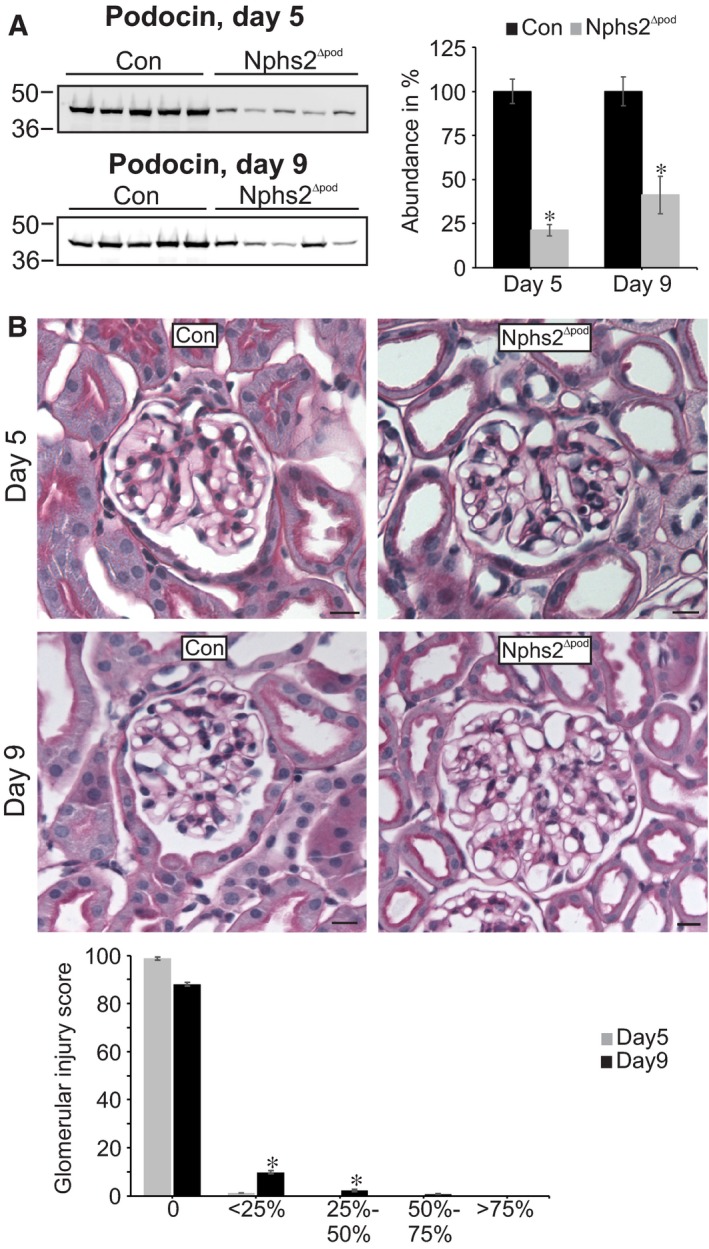
Assessment of podocin deletion and glomerular morphology during sodium retention. Western blots of podocin from membrane fractions of kidney cortex at 5 and 9 days (A). Densitometric evaluations are presented in the graph (right). Representative images of PAS‐stained sections from Con and Nphs2^∆pod^ at 5 and 9 days (B). No or only very mild glomerular and no tubule‐interstitial alterations were found at 5 and 9 days respectively. Magnifications scale bar = 20 µm. Results are arithmetic means ± SEM of n = 5 per group; *P < 0.05

**Figure 3:**
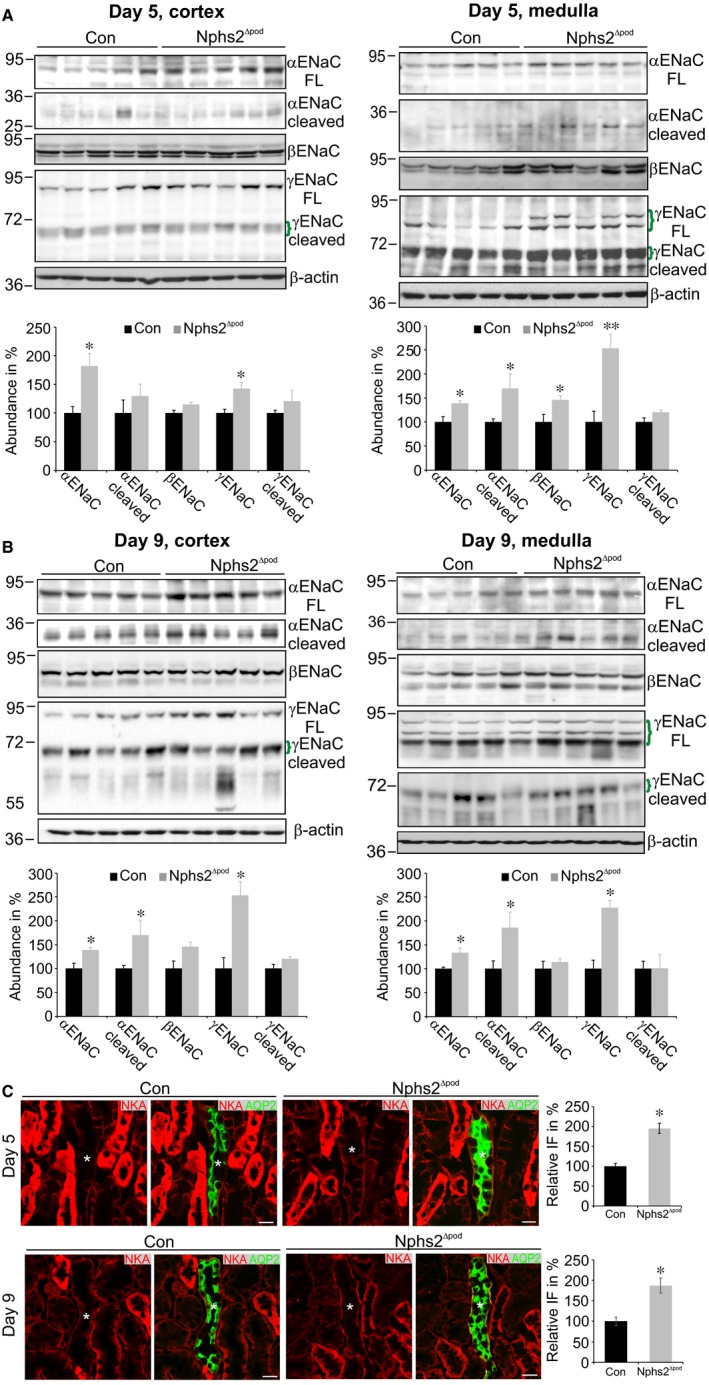
Assessment of ENaC subunit and collecting duct Na^+^/K^+^‐ATPase expression levels during sodium retention. Western blots of α‐, β‐ and γENaC from membrane fractions of kidney cortex and medulla at 5 (A) and 9 days (B). Specific bands are marked by green brackets. Densitometric evaluations are presented in the respective graphs below. Ponceau red staining and β‐actin served as loading control. Results are arithmetic means ± SEM of n = 5‐7 per group; *P < 0.05, **P < 0.005. Immunohistochemical double labelling of Na^+^/K^+^‐ATPase (red) and aquaporin‐2 (green) for the identification of collecting ducts (C). Collecting ducts are marked by an asterisk. Significantly increased collecting duct Na^+^/K^+^‐ATPase expression is encountered in Nphs2^∆pod^ at 5 and 9 days compared to control (Con) as depicted in the graph aside. Magnifications scale bar = 20 µm. Results are arithmetic means ± SEM of n = 5 per group; *P < 0.05

To analyse renal alterations in more detail, additional animal experiments at two time‐points after tamoxifen administration were chosen: day 5, during decreased sodium/creatinine ratio and day 9, when blood pressure starts to rise. Successful podocin deletion upon tamoxifen treatment was verified and confirmed (Figure 2A).Renal function analysis of mice at day 5 demonstrated reduced 24 hours sodium excretion, urinary Na/K ratio and fractional sodium excretion in Nphs2^∆pod^ compared to control (Table 1). Urinary albumin excretion started to rise but did not reach statistical significance. At day 9, albuminuria and proteinuria developed significantly (although at still very low levels) and fractional sodium excretion remained low in Nphs2^∆pod^ compared to control. Plasma aldosterone and vasopressin levels remained unchanged at time‐points of 5, 9 and 17 days of analysis. Next, morphological alteration of glomeruli and tubulointerstitium of Nphs2^∆pod^ were assessed by performing a semi‐quantitative analysis of PAS‐stained paraffin sections (Figure 2B) using a scoring system of 0‐4, where 0 = no damage and 4 = maximum score of damage encountered in 100% of the area analysed. At day 5, almost all glomeruli appear normal and a very small fraction showed marginal glomerular alterations. At day 9, mild alteration at some glomeruli was encountered fitting well with the significant increase in albuminuria. In the tubulointerstitium, no tubular alterations were found except for one tubular cast at day 9. These results demonstrate that glomeruli and tubulointerstitium are still intact at those time‐points, which is congruent with normal plasma creatinine and urea values. Because ENaC was shown to play a major role for sodium retention in other animal models with nephrotic or nephritic syndrome, cortical and medullary ENaC expression pattern was determined. Comparing Nphs2^∆pod^ to controls, Western blots of membrane fractions isolated from renal cortices and medullae at 5 days (Figure 3A) and 9 days (Figure 3B) revealed significantly increased expression of full‐length α‐ and γ‐ENaC subunit and cleaved αENaC fragments at 30 kDa. To address the question whether the occurrence of cleaved αENaC is due to increased overall αENaC levels or the result of augmented proteolytical cleavage, we calculated the ratio of cleaved α‐ and γENaC in relation to its full‐length subunit. Increased cleaved αENaC was observed in medulla at 5, 9 and 17 days (Table 2). The augmented expression level of full‐length α‐ and γENaC was not due to transcriptional alteration, as mRNA of α‐ and γENaC at 5 and 9 days did not change (Figure S1). Because apical Na^+^ entry is tightly coupled to the basolateral Na^+^ extrusion, we determined the basolateral α‐subunit Na^+^/K^+^‐ATPase (αNKA) expression level by measuring fluorescence intensity levels of cortical collecting ducts identified by aquaporin‐2 expression (Figure 3C). Semi‐quantified αNKA abundance from micrographs similar to (Figure 3C) after correction for background signal, cell area, and normalization to control values revealed significantly increased αNKA fluorescence at 5 and 9 days in Nphs2^∆pod^ compared to the controls. This demonstrates that augmented transepithelial sodium reabsorption may involve both apical ENaC and basolateral NKA.

**Table 1:** Renal functional data of control and Nphs2^∆pod^ during sodium retention at 5 and 9 days after podocin inactivation

	Day 5	Day 9	n
WT urine Na^+^/crea	36.9 ± 1.3	29.7 ± 2.3	5‐6
KO urine Na^+^/crea	27.7 ± 4.6*	32.0 ± 2.1	6‐7
WT urine Na^+^/K^+^	0.81 ± 0.07	0.68 ± 0.09	5‐6
KO urine Na^+^/K^+^	0.58 ± 0.08*	0.56 ± 0.07	6‐7
WT urine albumin (mg/L)	0.83 ± 0.3	1.17 ± 0.5	5‐6
KO urine albumin (mg/L)	5.12 ± 2.3	118.1 ± 5.4**	6‐7
WT urine protein (g/L)	1.36 ± 0.06	0.42 ± 0.8	5‐6
KO urine protein (g/L)	2.11 ± 1.12	4.88 ± 1.7**	6‐7
WT Fractional Na^+^ excretion, FE_NA _(%)	0.37 ± 0.03	0.34 ± 0.03	5‐6
KO Fractional Na^+^ excretion, FE_NA_ (%)	0.27 ± 0.03*	0.22 ± 0.02*	6‐7

Results are arithmetic means ± SEM of n = 5‐7 per group.

* *P* < 0.05,

** *P* < 0.01.

**Table 2:** Ratio of cleaved α‐or γENaC in relation to its full‐length subunit. Calculated ratio of the abundance of cleaved ENaC in relation to its full‐length subunit of cortical and medullary membrane fractions at 5, 9 and 17 days. Increased ratio of cleaved αENaC is found in the medulla at all time‐points analysed

	Cortex	Medulla
5 days		
αENaC cl/αENaC fl	0.71	1.23
γENaC cl/γENaC fl	0.85	0.47
9 days		
αENaC cl/αENaC fl	0.84	1.39
γENaC cl/γENaC fl	0.73	0.44
17 days		
αENaC cl/αENaC fl	0.94	1.98
γENaC cl/γENaC fl	1.05	1.06

### 3.3 Assessment of Nphs2^∆pod^ at day 17

Additionally, we performed an animal experiment using control and Nphs2^∆pod^ which we have stopped at 17 days after tamoxifen administration for renal analysis at a later time‐point when NS was fully developed. Plasma analysis of Nphs2^∆pod^ compared to control revealed reduced albumin and protein concentrations and increased creatinine levels (Table S2). Twenty‐four hours urine analysis of Nphs2^∆pod^ compared to control revealed increased albumin and protein excretion, reduced creatinine clearance and increased blood pressure. Ascites was regularly encountered in Nphs2^∆pod^ in comparison to its control. Morphologically, glomeruli demonstrated pronounced podocyte hypertrophy, mesangial matrix deposition and tubular proteinous casts (Figure S2A). Glomerular injury scoring showed that the majority of glomeruli is still normal; however, the amount of glomeruli appearing with severe alterations increased significantly (Figure S2B). Then, we performed Western blot analysis of membrane fractions from renal cortex and medulla of Nphs2^∆pod ^compared to control showing significantly increased ENaC subunit and cleaved α and γENaC expression. This is in agreement with various previously published results on volume handling in NS. Additionally, we performed double labelling of NKA with aquaporin‐2 to determine NKA fluorescence intensity levels in cortical collecting ducts, which were significantly increased at this time‐point (Figure 2D and 2).

### 3.4 Effects of amiloride in the nephrotic syndrome development

**Figure 4:**
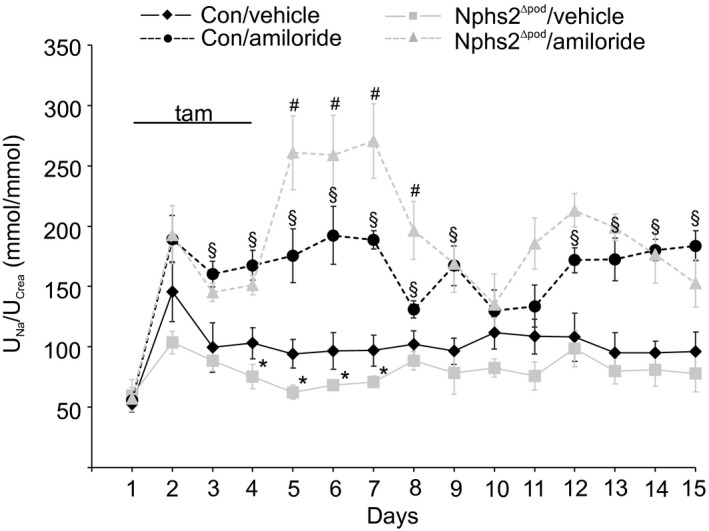
Course of urinary sodium/creatinine ratio from Con and Nphs2^∆pod^ treated with amiloride. Daily urinary sodium/creatinine ratio of control (Con) and Nphs2^∆pod^ treated with vehicle or amiloride (10 µg/g ip) during 14 days. Urine was collected 4 hours after injection of vehicle or amiloride. tam indicates the days of tamoxifen injection to induce Nphs2 knockout. Results are arithmetic means ± SEM of n = 5‐7 per group; *P < 0.05 Nphs2^∆pod^/vehicle versus control/vehicle; ^#^ *P* < 0.05 Nphs2^∆pod^/amiloride versus control/amiloride; and ^§^ *P* < 0.05 control/amiloride versus control/vehicle

To see and prove whether ENaC is activated and responsible for the reduced urinary sodium/creatinine ratio early in the NS development, an additional animal experiment was conducted with amiloride (Figure 4). As shown before, compared to their controls Nphs2^∆pod^/vehicle demonstrated significantly reduced urinary sodium/creatinine ratio between day 4 and day 7. Administration of amiloride to control increased urinary sodium/creatinine ratio at baseline and strongly augmented in Nphs2^∆pod^/amiloride between day 5 and day 8, proving increased ENaC activity early in NS development.

### 3.5 Urinary protease excretion

**Figure 5:**
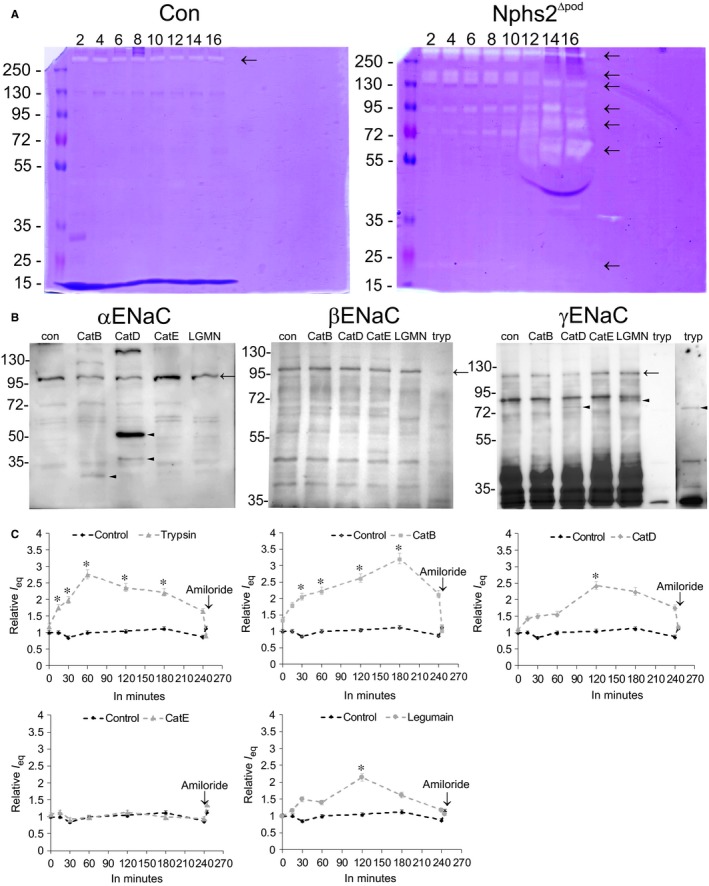
Presence of urinary protease activity, in vitro protease assays and proteolytic ENaC activation. Representative zymograms of urine loaded in normalization to urinary creatinine from control (Con) and Nphs2^Δpod^ obtained from day 2 to day 16 (A). Arrows mark protease‐positive bands. B, Western blots of protease assays using αENaC‐GST, βENaC‐GST or γENaC‐GST incubated either with cathepsin B (CatB), cathepsin D (CatD), cathepsin E (CatE) or legumain (LGMN). In the case of β‐ and γENaC trypsin (tryp) was used as a positive control. In the case of γENaC‐GST, to observe the trypsin cleaved band, Western blot exposure time was increased (extract on the right). C, Equivalent short circuit current of mpkCCD_cl14_ monolayers incubated either with trypsin, which served as positive control, or CatB, CatD, CatE or legumain over a time period of 240 min. Results are means ± SEM of n = 3 per group per experiment. Experiments were repeated four times, *P < 0.05

Because of the early αENaC processing already at 5 days after FSGS induction, we analysed the urine of Nphs2^∆pod^ and their controls from day 2 until day 16. In controls, a zymogram‐positive band was observed at above 260 kDa, which also occurred in the urine of Nphs2^∆pod^ with a constant intensity over the time (Figure 5A). Surprisingly, already at day 2 proteolytic activity was observed in the urine of Nphs2^∆pod^. With time, intensity of zymogram‐positive bands increased as well as the number of proteases running at different molecular weight. At 2 days, a band at 140‐160 kDa was found, at day 6 an additional band at 100 kDa was observed, at day 8 a band at 70 kDa was observed, and at days 10 and 12 bands at 85 kDa and 60 kDa was found respectively. Additionally, a band at 25 kDa from day 2 to 6 was observed. Then, we aimed to identify the protease responsible for the proteolysis of ENaC. Therefore, the urine of Nphs2^∆pod^ from day 2 until day 9 was collected, HPLC‐purified and the resulting 80 fraction tested on their proteolytic activity by gel zymography. Eleven fractions were found to be positive and neighbouring fractions with the same molecular weight were pooled. Fractions 8‐9, 10‐11, 12‐13, 14‐15, 16 and 17‐18 were tested for their ability to augment ENaC activity. Fractions 8‐9, 10‐11, 12‐13 and 16 significantly increased amiloride‐sensitive equivalent transepithelial currents in mpkCCD_cl14_ cells (data not shown). All six protease‐positive HPLC fractions were analysed by liquid chromatography (LC)‐tandem mass spectrometry (MS/MS) and identified proteases are summarized in Table 3. The identified proteases were typically present in multiple HPLC fractions; hence they are summarized as a joint list.

**Table 3:** Proteases identified from HPLC‐purified urine of Nphs2^∆pod^ from day 2 until day 9 after podocin inactivation. Protein name, gene name and protein ID of the identified proteases are listed. Lysosomal proteases are marked in bold and known γENaC cleaving proteases are marked in cursive

Protein name	Gene name	Protein ID
Hepatocyte growth factor activator	Hgfac	Q9R098
Napsin‐A	Napsa	O09043
**Legumain**	Lgmn	O89017
Complement factor D	Cfd	P03953
Complement factor B	Cfb	P04186
Lactotransferrin	Ltf	P08071
Angiotensin‐converting enzyme	Ace	P09470
**Cathepsin B**	Ctsb	P10605
*Kallikrein‐1*	Klk1	P15947
Glutamyl aminopeptidase	Enpep	P16406
**Lysosomal protective protein**	Ctsa	P16675
**Cathepsin D**	Ctsd	F6Y6L6
*Prothrombin*	F2	H7BX99
*Plasminogen*	Plg	P20918
Meprin A subunit alpha	Mep1a	P28825
**Cathepsin E**	Ctse	D3Z6T3
Aminopeptidase N	Anpep	P97449
Complement factor I	Cfi	Q61129
Haptoglobin	Hp	Q61646
Meprin A subunit beta	Mep1b	Q61847
Lysosomal Pro‐X carboxypeptidase	Prcp	Q7TMR0
Serotransferrin	Tf	Q921I1
Dipeptidyl peptidase 2	Dpp7	Q9ET22
Acid ceramidase	Asah1	Q9WV54
Mast cell protease‐11	Prss34	Q80UR4
*Prostasin*	Prss8	Q99L44

### 3.6 ENaC subunit processing and activation by lysosomal enzymes

Based on our results, we were focusing on αENaC cleaving enzymes and identified cathepsin B (see Table 3), which was shown previously to activate ENaC currents by proteolytical processing of αENaC.^19^ We have been suggested that there maybe also other lysosomal enzymes, as found and highlighted in Table 3, may be able to cleave αENaC. Performing in vitro protease assays using αENaC‐GST fusion proteins, we found cathepsin B cleaved αENaC at the known ‘furin’ cleavage site with a resulting product at 30 kDa (Figure 5B). Cathepsin D cleaved αENaC at an unknown site with resulting products at approx. 38 and 50 kDa. Cathepsin E and legumain were unable to process αENaC. Incubating γENaC‐GST fusion protein with lysosomal enzymes showed that cathepsin D faintly cleaved γENaC at the appropriate site with a resulting product at 70 kDa, see positive control using trypsin. Legumain processed γENaC at approx. 72 kDa. Cathepsins B and E were unable to process γENaC. Incubating βENaC‐GST fusion proteins with lysosomal enzymes did not result in proteolytical processing, demonstrating the specificity of lysosomal enzymes for a specific subunit. Next, we tested the ability of the lysosomal enzymes to augment ENaC activity. Incubating mpkCCD_cl14_ cell monolayers with cathepsin B resulted in a significant augmentation of equivalent transepithelial currents within 30 minutes (Figure 5C). Cathepsin D and legumain increased equivalent transepithelial currents after 2 hours. For comparison, trypsin increased equivalent transepithelial current within 15 minutes. Cathepsin E did not alter the equivalent transepithelial current.

### 3.7 Increased cathepsin B expression level in Nphs2^∆pod^

**Figure 6:**
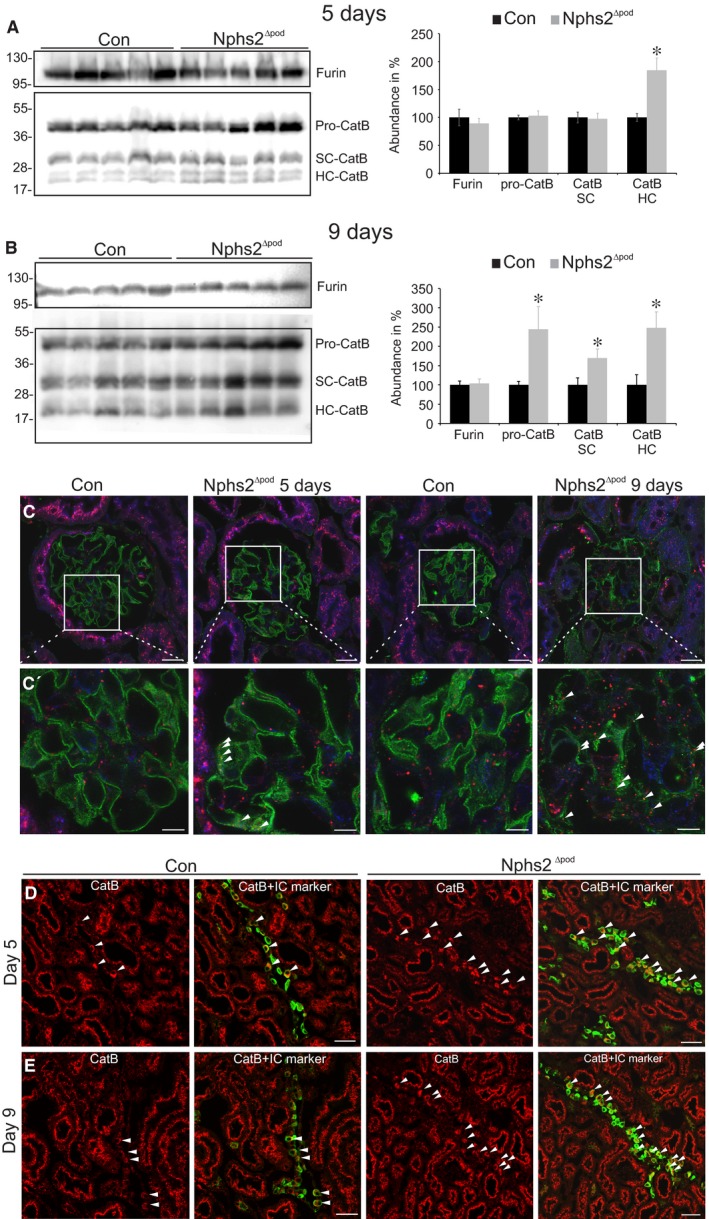
Furin and cathepsin B expression during sodium retention. Western blots and densitometrical evaluation of furin and cathepsin B occurring as proenzyme (proCatB), as active enzyme single chain (SC‐CatB) or heavy chain (HC‐CatB) from renal membrane fractions of control (Con) and Nphs2^∆pod^ at 5 days (A) and 9 days (B). Results are arithmetic means ± SEM of n = 5 per group; *P < 0.05. C and C’, Merge images of immunohistochemical triple labelling using cathepsin B (red), lysosomal marker Lamp‐1 (blue) and podocyte maker nephrin (green), in control (Con) and Nphs2^∆pod^ at 5 days and 9 days. Magnification scale bar = 20 µm. C’, Magnification of insert. Arrowheads mark strong CatB expression in podocytes. Magnification scale bar = 10µm. D and E, Immunohistochemical double labelling of cathepsin B (CatB, red) and intercalated cell marker β1‐subunit of the V‐ATPase (green) in control and Nphs2^∆pod^ at 5 days (C) and 9 days (D). Arrowheads mark strong CatB expression in intercalated cells. Magnifications scale bar = 50 µm

Next, the proteases furin, cathepsin B and D were analysed on their expression level. Western blot analysis of cathepsin B revealed significantly augmented expression in Nphs2^∆pod^ compared to their controls at 5 and at 9 days after tamoxifen administration (Figure 6A and 6 respectively). Furin and cathepsin D remained unaltered (results for cathepsin D are not shown). For cellular analysis, immunohistochemical staining of cathepsin B was performed. Triple labelling of cathepsin B, lysosomes and podocytes after 5 days revealed increased vesicular cathepsin B expression in podocytes and after 9 days, it overall increased vesicular cathepsin B staining within podocytes and glomerulus (Figure 6C and 6’). In the proximal tubule, cathepsin B was observed in lysosomes. Surprisingly, a strong increase in cathepsin B in intercalated cells was detected at 5 and at 9 days (Figure 6D and 6). Additionally, we followed the hypothesis that increased proximal tubular albumin uptake may activate endocytosis and that lysosomal enzymes may appear in the urine upon lysosomal spill over. Therefore, we determined endocytosed albumin in proximal tubular profiles, identified by double labelling with megalin (Figure S3A). At 5 days, no difference in albumin fluorescence was observed between Nphs2^∆pod^ and control. At 9 days and more pronounced at 17 days, endocytosed albumin increased significantly. As sodium retention occurs between days 4‐7 it seems unlikely, that increased urinary cathepsin B stems from the proximal tubular lysosome spill over. Additionally, we performed coomassie staining of SDS‐PAGE and Western blots from daily spot urine loaded after creatinine adjustment (Figure S3B). Albuminuria was starting at days 4‐6 and unselective gross proteinuria on days 13‐14. Western blots of cathepsin B showed increased active sc‐cathepsin B expression early between day 4 and 7, whereas plasminogen and plasmin started to increase at days 13‐14. We also tested whether albumin may activate cathepsin B expression in cortical collecting duct cells and incubated mpkCCD_cl14_ cells with albumin at concentrations ranging from 0 to 20 mg/mL for 6 hours and at 10 mg/mL for 0, 3, 6 and 24 hours; however, no significant changes were observed (data are not shown).

### 3.8 Impacts of cathepsin B inhibitor CA‐074Me in Nphs2^∆pod^

To analyse whether cathepsin B may impact the disease progression in Nphs2^∆pod^, an additional animal experiment was performed with control and Nphs2^∆pod^ receiving either CA‐074Me (a membrane‐permeable cathepsin B inhibitor) or vehicle for 14 days starting on the day after the first tamoxifen injection. As expected at day 9, Nphs2^∆pod^ receiving vehicle developed significantly higher blood pressure, which remained high until the end of the experiment at 14 days (Figure S4). Blood pressure of Nphs2^∆pod^ receiving CA‐074Me, however, remained at control levels.

## 4 DISCUSSION

Proteinuria, volume retention and subsequent hypertension and/or oedema are hallmarks of the NS and possible mechanisms have been identified previously. The murine model of targeted podocin gene inactivation was shown to develop the entire characteristics of FSGS with NS until the fourth week after induction^3^ allowing us to carefully examine renal function in a time‐dependent manner over a longer time period then in animal models of PAN‐induced nephrosis or doxorubicin‐induced NS.^10^, ^14^, ^16^, ^17^ We have observed sodium retention, hypertension and gross/unselective proteinuria to occur in a chronologic successive order. Focusing on the time‐point of sodium retention between day 4 and 7 and start of increased blood pressure at day 9 after tamoxifen administration, analysis of ENaC expression pattern was surprising. Increased occurrence of cleaved αENaC fragments and abundance of full‐length ENaC subunits without changes in aldosterone and vasopressin levels were observed. An increased ENaC abundance was found previously in many other proteinuric animal models.^6^, ^7^, ^8^, ^9^ Augmented ENaC function, however, seems to be unrelated to hormonal stimulation, as various hormonal blockades did not change the clinical outcome.^7^, ^10^ Transcriptional regulation can also be excluded as ENaC mRNA expressions levels neither vary at 5 nor at 9 days, similarly as previously reported.^20^ In Nphs2^∆pod^, the augmented ENaC expression levels and or αENaC cleavage at 5 and 9 days is of functional relevance because NKA expression levels from the cortical collecting duct are consistently increased compared to control and other nephron segments in the same section. Supporting evidence for early increased ENaC activity was gained from daily administration of amiloride to control and Nphs2^∆pod^. Highest plasma values for amiloride occur 3‐4 hours after amiloride administration, the time‐point where sodium was determined from spot urine. From our experiment, we observed that between day 5 and 9 before sodium/creatinine ratio increased in control/amiloride and augmented strongly in Nphs2^∆pod^/amiloride suggesting that using this application procedure sodium/creatinine ratio mirrors ENaC channel activity. These results support the overfill hypothesis where sodium retention is related to an intrinsic renal defect in sodium handling.

With disease progression, proteinuria establishes, plasma albumin values decrease and oedema can be regularly encountered at 17 days of analysis. Those later changes support rather the under fill hypothesis where decreased plasma oncotic pressure by hypoalbuminemia and fluid shifts from intravascular to the interstitial compartment can be found.

The appearance of αENaC fragments at 5 days suggests the existence of local proteases; therefore, the urine was tested for proteolytic activity. Already at the second day of animal experiment, elevated levels of proteases can be found in the urine of Nphs2^∆pod^ which constantly increase with the time. From HPLC‐purified urine of Nphs2^∆pod^, we identified known γENaC cleaving proteases, such as prostasin^20^, ^21^ kallikrein‐1^12^, ^22^, ^23^ plasminogen and prothrombin.^12^ Additionally, several lysosomal proteases, among them cathepsin B, ‐D, ‐E and legumain were encountered. Focusing on putative αENaC cleaving enzymes, we could confirm the results of A. Alli et al^13^ showing proteolytic αENaC processing by cathepsin B resulting in increased relative currents in mpkCCD_cl14_ cells. Among other lysosomal proteases only cathepsin D cleaved αENaC; however, at a yet unknown site leading to 50 and 40 kDa fragments. Whether cathepsin D cleavage is of functional relevance needs to be identified in future. Additionally, we tested whether the identified lysosomal proteases cleave αENaC. Using trypsin as positive control, we observed a very mild processing by cathepsin D and legumain by in vitro protease assays. Although Tan et al^24^ reported that cathepsin B cleaves αENaC and γENaC, we did not observe γENaC processing which may be due to differences in techniques and models used. The very faint cleavage of γENaC by cathepsin D at 70 kDa and legumain at 72 kDa corresponds to the increase in relative currents in mpkCCD_cl14_ cells at 2 hours. A more detailed analysis for further exploration needs to be performed in future.

In Nphs2^∆pod^ during the time of early sodium retention between day 5 and 9, overall glomerular morphology remains largely intact, the glomerular filter however is altered and shows albuminuria/selective proteinuria. Therefore, plasma proteases as shown for plasminogen are still unable to pass and do not account for the sodium retention during this period assuming that urinary proteases identified might stem from the kidney itself. Lysosomal cathepsin B activation and proximal tubular spill over into the primary ultrafiltrate was postulated to account for increased urinary cathepsin B activity in proteinuric diseases.^25^ However, analysis of endogenous expression of taken‐up albumin and β2‐microglobulin, as a low‐molecular weight protein, did not change at five days and was slightly increased at 9 days (data not shown);indicating that it cannot account for increased cathepsin B activity at least at 5 days. Furthermore, we tested whether albumin may augment cathepsin B expression levels; however, no obvious alterations were observed. Immunohistochemical localization of cathepsin B revealed strong expression in proximal tubular profiles and to lesser extent in the glomerulus, distal tubule and collecting duct system. Because of the high proximal tubular levels of endogenous cysteine protease inhibitors, cystatin C,^26^ proximal tubular‐derived cathepsin B may not contribute much to the increased renal expression levels observed in Western blots. In Nphs2^∆pod^ at 5 and 9 days, increased cathepsin B expression was found in glomeruli and more strongly in intercalated cells; therefore, we assume that either glomerulus‐derived cathepsin B via ultrafiltrate or intercalated cell‐derived cathepsin B paracrine reach the principal cells for αENaC cleavage and therefore ENaC channel activation. The important role of cathepsin B in renal disease progression was demonstrated recently showing that cathepsin B knockout mice were more resistant and recovered faster after glomerular damage upon podocyte injury induced by nephrotoxic serum.^27^

In summary, using the Nphs2^∆pod^ mice, we were able to identify the chronology of the development of hallmarks of the NS. Early after genetic *Nphs2* deletion, sodium retention occurred, followed by hypertension and gross proteinuria. This early sodium retention is based on augmented ENaC activity through proteolytical processing of αENaC. Cathepsin B was identified as αENaC cleaving enzyme where its blockade prevented hypertension.

## CONFLICT OF INTEREST

There is no conflict of interest.
