# Supplementary material for: Cathepsin B increases ENaC activity leading to hypertension early in nephrotic syndrome
Source: J Cell Mol Med. 2019 Jul 31;23(10):6543–53. doi: 10.1111/jcmm.14387 (PMC6787568; doi:10.1111/jcmm.14387)
Supplement: Supplementary file 5 [file JCMM-23-6543-s005.docx]

Supplementary Table 1

|  | 17 day | n |
| --- | --- | --- |
|  |  |  |
| **WT urine [ ml/24h]** | 0.63 ± 0.05 | 5-6 |
| **KO urine [ ml/24h]** | 0.59 ± 0.08 | 6-7 |
|  |  |  |
| **WT BW [g]** | 23.6 ± 3.5 | 5-6 |
| **KO BW [g]** | 20.4 ± 2.5 | 6-7 |
|  |  |  |
| **WT plasma Na^+^ [ mM/l]** | 152.8 ± 1.4 | 5-6 |
| **KO plasma Na^+^ [ mM/l]** | 149.1 ± 0.8 | 6-7 |
|  |  |  |
| **WT plasma K^+^ [ mM/l]** | 8.7 ± 0.8 | 5-6 |
| **KO plasma K^+^ [ mM/l]** | 9 .3 ± 0.7 | 6-7 |
|  |  |  |
| **WT plasma Cl^-^ [ mM/l]** | 82.0 ± 0.5 | 5-6 |
| **KO plasma Cl^-^ [ mM/l]** | 81.6 ± 1.7 | 6-7 |
|  |  |  |
| **WT plasma albumin [mg/l]** | 24.7 ± 1.0 | 5-6 |
| **KO plasma albumin [mg/l]** | 11.3 ± 2.9 *** | 6-7 |
|  |  |  |
| **WT plasma protein [g/l]** | 47.7 ± 1.1 | 5-6 |
| **KO plasma protein [g/l]** | 36.1 ± 2.1*** | 6-7 |
|  |  |  |
| **WT plasma urea [ md/dl]** | 19.4 ± 1.3 | 5-6 |
| **KO plasma urea [ md/dl]** | 24.9 ± 3.7 | 6-7 |
|  |  |  |
| **WT plasma creatinine [ uM/l]** | 15.8 ± 3.6 | 5-6 |
| **KO plasma creatinine [ uM/l]** | 27.3 ± 2.0* | 6-7 |
|  |  |  |
| **WT plasma aldosterone [ pg/ml]** | 424.8 ± 67 | 5-6 |
| **KO plasma aldosterone [pg/ml]** | 512.1 ± 118 | 6-7 |
|  |  |  |
| **WT plasma vasopressin [ pg/ml]** | 1.15 ± 0.60 | 5-6 |
| **KO plasma vasopressin [pg/ml]** | 1.13 ± 0.43 | 6-7 |
|  |  |  |
| **WT urine Na^+^ [mM/l/24h]** | 100 ± 6.4 | 5-6 |
| **KO urine Na^+^ [mM/l/24h]** | 110 ± 1.6 | 6-7 |
|  |  |  |
| **WT urine K^+^ [mM/l/24h]** | 130.2 ± 12.7 | 5-6 |
| **KO urine K^+^ [mM/l/24h]** | 124.6 ± 22.3 | 6-7 |
|  |  |  |
| **WT urine Cl^-^ [mM/l/24h]** | 93.9 ± 15.5 | 5-6 |
| **KO urine Cl^-^ [mM/l/24h]** | 96.7 ± 17.7 | 6-7 |
|  |  |  |
| **WT urine Na^+^ / K ^+^ [mM/l/24h]** | 0.70 ± 0.05 | 5-6 |
| **KO urine Na^+^/ K ^+^ [mM/l/24h]** | 0.80 ± 0.09 | 6-7 |
|  |  |  |
| **WT urine albumin [mg/l/24h]** | 6.0 ± 2.8 | 5-6 |
| **KO urine albumin [mg/l/24h]** | 134.8 ± 19.1*** | 6-7 |
|  |  |  |
| **WT urine protein [g/l/24h]** | 0.49 ± 0.1 | 5-6 |
| **KO urine protein [g/l/24h]** | 40.3 ± 12.1* | 6-7 |
|  |  |  |
| **WT urine osmolarity [mOsmol/ kg/24h ]** | 1448 ± 243 | 5-6 |
| **KO urine osmolarity [mOsmol/ kg/24h]** | 1530 ± 262 | 6-7 |
|  |  |  |
| **WT Fractional Na^+^ excretion , FE_NA_ (%)** | 0.33 ± 0.08 | 5-6 |
| **KO Fractional Na^+^ excretion , FE_NA_ (%)** | 0.42 ± 0.07 | 6-7 |
|  |  |  |
| **WT Creatinine clearance GFR (µl/min/BW)** | 109.0 ± 28.8 | 5-6 |
| **KO Creatinine clearance GFR (µl/min/BW)** | 30.4 ± 7.5* | 6-7 |
|  |  |  |
| **WT systolic bp [mm Hg]** | 79 ± 3 | 5-6 |
| **KO systolic bp [mm Hg]** | 107 ± 3*** | 6-7 |
|  |  |  |
| **WT diastolic bp [mm Hg]** | 59 ± 2 | 5-6 |
| **KO diastolic bp [mm Hg]** | 85 ± 2*** | 6-7 |
