# Supplementary material for: Cathepsin B increases ENaC activity leading to hypertension early in nephrotic syndrome
Source: J Cell Mol Med. 2019 Jul 31;23(10):6543–53. doi: 10.1111/jcmm.14387 (PMC6787568; doi:10.1111/jcmm.14387)
Supplement: Supplementary file 6 [file JCMM-23-6543-s006.docx]

**Supplemental material**

***Functional Measurements***

To study renal function mice were placed in metabolic cages every day for 4 hours for a time of 4 weeks or 14 days in the second set of animal experiment starting one day before tamoxifen induction. Mice received standard chow diet and tap water. Additionally, mice were weighed and blood pressure was determined by non-invasive blood pressure measurements via tail- plethysmography cuff method using the IITC Life Science MRBP Blood Pressure System every day in an observer-blinded fashion. Mice were kept in a restrainer, which was placed in the Blood Pressure System platform. Measurements were started when the temperature of the platform reached 30ºC or 32ºC (according to the MRBP Blood Pressure System for mice operating manual). Mice were quiet and relaxed during measurements. Each measure was repeated 3 times to define the average level of systolic and diastolic blood pressure. Urinary electrolytes, creatinine and albumin were measured using standardized methods of the hospital laboratory, Fribourg Switzerland. Additionally, mice at 5, 9 and 17 days of induction were placed for 24h in metabolic cages for urine collection. Retroorbital blood sampling was performed afterwards and before *in vivo* perfusion fixation. Renal functional parameters were determined from blood and urine samples at the Fribourg Cantonal Hospital (Service Laboratoir HFR) using an ion-selective electrode indirect method for detection of Na^+^, K^+^, Cl^-^, Cat.No. 0588392001 (ISE indirect Na^+^, K^+^, C^-^ for Gen.2, Roche, Basel Switzerland); quantitative determination of creatinine, kit CREJ2- Cat. No. 04810716 190 (Creatinine Jaffé Gen.2**,** Roche, Basel Switzerland); quantitative determination of urinary protein using TPUC3, Cat. No. 03333825 190 (Total Protein Urine/CSF Gen.3, Roche, Basel Switzerland); quantitative determination of albumin ALBT2, Cat. No. 04469658 190 (Tina-quant Albumin Gen.2, Roche, Basel Switzerland). Fractional sodium excretion (FE_Na_) and glomerular filtration rate (GFR) were calculated using standard equations. 1

***Cell Culture experiments and equivalent short circuit current studies***

Cortical collecting ducts mpkCCD_cl14_ cells were cultured in defined medium as described previously [1]. In brief, growth medium was composed of equal volumes of Dulbecco's modified Eagle's medium and Ham's F12, 60 nmol/l Na+ selenate, 5 μg/ml transferrin, 50 nmol/l dexamethasone, 1 nmol/l triiodothyronine, 10 ng/ml epidermal growth factor, 5 μg/ml insulin, 2% fetal bovine serum, and 100 μg/ml penicillin/streptomycin. Cells were grown in a 5% CO2/ 95% air atmosphere at 37°C. Cells were grown in two chamber devices (Millicell, Millipore, Schaffhausen, Switzerland), starved overnight in FCS-, dexamethasone and insulin free medium followed by incubation with HPLC fractions or 10 µg/ml of the active lysosomal proteases cathepsin B, - D, -E or legumain for 30 min at pH = 6.5. Then cellular voltage and resistance was determined using an epithelial Volt-Ohm Meter (Millicell ERS-2, Millipore, Schaffhausen, Switzerland) during 4 hours. The equivalent short circuit current across the mpkCCD_cl14_ cell monolayer was calculated using Ohm’s law as the quotient of transepithelial voltage (V*_T_*) to transepithelial electrical resistance (R*_T_*) under open circuit conditions. To determine the amiloride-sensitive current, 10 µM amiloride (SigmaAldrich) was added to the apical cell surface at the end of the experiments and voltage and resistance were measured immediately.

**Quantitative PCR using TaqMan probes**

RNA was isolated from kidneys according to manufacturer´s instruction (NucleoSpin RNA/protein, Machery & Nagel). 2 µg RNA were reverse transcribed into cDNA in the presence of M-MLV reverse transcriptase (Promega), dNTP and random hexamer primer. cDNA was added to TaqMan Fast Advanced master mix (Applied Biosystem, 4444556), TATA-box binding protein (Tbp) (Applied Biosystem 4319413E), SCNN1a (Mm00803386_m1) or SCNN1g (Mm00441228_m1) and subjected to qPCR.

***Fixation and tissue processing for immunohistochemistry and immunoblotting***

Kidneys were shock-frozen for biochemical evaluation or perfused retrogradely using 4% PFA in PBS and either postfixed over night and subsequently transfered into 0.1% PFA in PBS for paraffin embedding or transferred into 800 mOsm Sucrose in PBS overnight and subsequently frozen in OCT. Paraffin embedded sections were further processed for PAS-staining. Brightfield imaging was performed on a Leica DMRE light microscope and image processing using Metavue software (Molecular Devices). For isolation of membrane fractions kidneys were homogenized in isolation buffer and processed as described [2]. Total protein concentration was measured using the Pierce BCA Protein Assay reagent kit (ThermoFischer Scientific, Reinach, Switzerland) and controlled by Coomassie staining.

***Morphological assessment of FSGS development***

Periodic acid-Schiff (PAS) stained 5 µm paraffin sections were studied to evaluate the degree and extent of glomerulosclerosis on day 5, 9 and 17 after FSGS induction. Signs for glomerular damage comprised podocyte hypertrophy, mesangial matrix expansion, collapsed capillaries and pseudocrescent formation. For the tubulointerstitial injury characterization tubular dilation and atrophy, necrosis and proteineous casts were assessed. A semi-quantitative score was applied as described in Theilig et al. [3] and performed in an observer blinded fashion.

***SDS-PAGE and Immunoblotting***

Proteins were solubilized and SDS gel electrophoresis was performed on 8-10% polyacrylamide gels. After electrophoretic transfer of the proteins to nitrocellulose membranes, equity in protein loading and blotting was verified by membrane staining using 0.1% Ponceau red. Membranes were probed with primary antibodies and then exposed to HRP-conjugated secondary antibodies (Dianova, Hamburg, Germany). Immunoreactive bands were detected by chemiluminescence (Amersham Pharmacia, Glattbrugg, Switzerland). Densitometric evaluation was performed by Syngene (G:Box, Chemi XX6). In the case of γENaC proteins were deglycosylated by using PNGase F (New England Biolabs). Resulting values were normalized to β-actin values and are presented in percent of control values obtained from the control group.

***Immunohistochemistry***

Cryosections were blocked with 5% skim milk/PBS, incubated with the respective primary antibody followed by the suitable cy-2 or cy-3-coupled secondary antibody (Dianova). Double-antibody staining procedure was controlled by parallel incubation of consecutive sections, each probed only with one single antibody. Sections were analyzed using a multilaser confocal scanning microscope (SP5, Leica, Heerbrugg, Switzerland).

***Antibodies***

The following antibodies were used: rabbit anti-αENaC, rabbit anti-βENaC, rabbit anti-γENaC [4], mouse anti-β-actin (SigmaAldrich, Buchs, Schweiz), goat anti-albumin (Bethyl Laboratories), guinea pig anti-megalin [3] rabbit anti-furin (Proteintech, Germany), mouse-anti Na^+^/K^+^-ATPase (MerckMillipore, Germany), Alexa647-coupled phalloidin (ThermoFischer Scientific, Zug, Switzerland), goat anti-Cathepsin B (R and D Systems), rabbit anti-LAMP-1 (Santa Cruz Biotechnology), guinea pig anti-nephrin (Progen, Heidelberg Germany), rabbit anti-plasminogen (Abcam), anti-β1V-ATPase (generous gift of C. Wagner, Zürich, Switzerland), rabbit anti-podocin (P0372, SigmaAldrich).

***Measurements of Na^+^/K^+^-ATPase and albumin relative fluorescence unit***

The relative fluorescence intensity was measured using the LASAF software (Leica, Heerbrugg, Switzerland). For the measurement of Na^+^/K^+^-ATPase fluorescence intensities, regions of interest (ROI) were placed around cortical collecting duct profiles identified by aquaporin-2 staining within the renal cortex. For the measurement of albumin fluorescence intensities, regions of interest (ROI) were placed around proximal tubular profiles identified by megalin staining within the renal cortex. The relative fluorescent unit (rfu) values were calculated for each tubule after image background subtraction (fluorescence intensity of regions without cells)*.* All tubules per section per animal were analyzed.

***HPLC purification***

24 hours urine from 5 *Nphs2*^Δpod^ mice obtained from day 2 until day 9 were pooled and purified by size chromatography using sephadex column HiLoad 16/600 Superdex 200pg (GE Healthcare).

***Proteolytical activity measurements***

To examine urinary protease activity, gelatin zymography (ThermoFisher Scientific) was performed using urine samples obtained from control and Nphs2^Δpod^ mice normalized to 1 µg creatinine (n = 5) and of HPLC fractions.

***Label-free urinary proteome analysis***

Proteins of HPLC fractions obtained of pooled 24 hours urine from Nphs2^Δpod^ mice were reduced and alkylated using 5 mM DTT and 20 mM Iodacetamide, precipitated with trichloroacetic acid (TCA) and digested with sequencing grade trypsin. Following clean-up with C18 stage tips, samples were analyzed on a Q-Exactive plus system (Thermo Scientific, Bremen, Germany) as described elsewhere [5]. For data analysis, MS files were analyzed by MaxQuant version 1.3.0.5 with the Uniprot mouse database downloaded in October 2014, counting 43393 entries. The identified proteins were cross-referenced with a list of all murine proteases downloaded from “MEROPS” [6] in 2014. Files obtained by MaxQuant were further processed using RStudio v.0.99.446 as an IDE for R (R Foundation for Statistical Computing, Vienna, Austria) as previously described [7].

***Recombinant protein production and in vitro protease assay***

ENaC subunits cloned into a pGEX4T-2 expression vector were used for the production of recombinant fusion proteins of αENaC, βENaC and γENaC and was performed as described by Alli et al.[8]. For in vitro protein assays, active cathepsin B purified from human liver (purity > 95%, Calbiochem), active cathepsin D purified from human liver (purity > 95%, SigmaAldrich), active human recombinant cathepsin E (purity > 90%; Biovision) and active human recombinant legumain (purity > 75%, Antibodies-Online) and trypsin (SigmaAldrich) were used. Five microgram of freshly prepared recombinant ENaC GST fusion protein was incubated in 50 mM Tris-HCl pH = 6.5 with 10 µg/ml of either cathepsin B, - D, -E or legumain for 4 hours at 37°C followed by Western blot analysis for detection of each ENaC subunit.

**Supplemetary Figures**

**Supplementary Fig. 1. Assessment of renal alterations after 17 days after Nphs2 knockout induction. (A)** At 17 days, approx. 40 % of glomeruli were visibly damaged and tubular protein casts within the papilla were found. Otherwise tubule-interstitial alterations were not observed. Magnifications scale bar = 20 µm. **(B)** Applying a semi quantitative score from 0 - 4, where 0 is no damage and 4 is 100% damage, even at 17 days, only moderate glomerular injury can be encountered. Results are means ± SEM of n = 5 per group; **P* < 0.05. **(C)** Western blot analysis of α-, β-, and γENaC from membrane fractions of kidney cortex and medulla. Densitometric evaluations are presented in the respective graphs below. Results are arithmetic means ± SEM of n = 5 per group; **P* < 0.05, ***P* < 0.05. Ponceau red staining and β-actin served as loading control. **(D)** Immunohistochemical double labeling of Na+/K+-ATPase (red) and aquaporin-2 (green) for the identification of collecting ducts. Collecting ducts are marked by an asterisk. Magnifications scale bar = 20 µm. **(E)** Semiquantified Na+/K+-ATPase abundance from micrographs similar to (D) after correction for background signal, cell area, and normalization to control values (n=4). * indicates significant changes relative to control.

**Supplementary Fig. 2. Proximal tubular albumin uptake and daily urinary protein, cathepsin B and plasminogen excretion. (A)** Immunohistochemical double labeling of albumin (red) and megalin (green) for the identification of proximal tubules. Magnifications scale bar = 20 µm. (Right) Semiquantified albumin abundance from micrographs similar to (A) after correction for background signal, cell area, and normalization to control values (n=4). * indicates significant changes relative to control. **(B)** (Left) Coomassie staining of urine samples subjected to SDS-PAGE from control (Con) and Nphs2^Δpod^ mice (Nphs2^Δpod^) from day 4 to 15. Sample volume loaded was according to 10µg creatinine. (Right) Representative western blots of cathepsin B and plasminogen excretion of Nphs2^Δpod^ mice from day 4 to 15. Active cathepsin B was found between 4 - 7 days and plasminogen (at ca. 120kDa) and plasmin (at ca. 85 kDa) from day 12 - 13 onward.

**Supplementary Fig. 3. Blockade of cathepsin B activity normalizes blood pressure in nephrotic syndrome.** Systolic **(A)** and diastolic **(B)** blood pressure of control and Nphs2^∆pod^ receiving either Ca-074Me (cathepsin B inhibitor) or vehicle for 14 days. Results are means ± SEM of n = 6 in Nphs2^∆pod^/ vehicle and Nphs2^∆pod^/ CA-074Me and n = 4 in control/ CA-074Me and control/vehicle; **P* < 0.05.

**Supplementary References**

1. Bens M, Vallet V, Cluzeaud F, Pascual-Letallec L, Kahn A, Rafestin-Oblin ME, Rossier BC, Vandewalle A (1999) Corticosteroid-dependent sodium transport in a novel immortalized mouse collecting duct principal cell line. J Am Soc Nephrol 10:923-934

2. Gadau J, Peters H, Kastner C, Kuhn H, Nieminen-Kelha M, Khadzhynov D, Kramer S, Castrop H, Bachmann S, Theilig F (2009) Mechanisms of tubular volume retention in immune-mediated glomerulonephritis. Kidney Int 75:699-710

3. Theilig F, Kriz W, Jerichow T, Schrade P, Hahnel B, Willnow T, Le Hir M, Bachmann S (2007) Abrogation of protein uptake through megalin-deficient proximal tubules does not safeguard against tubulointerstitial injury. J Am Soc Nephrol 18:1824-1834

4. Kastner C, Pohl M, Sendeski M, Stange G, Wagner CA, Jensen B, Patzak A, Bachmann S, Theilig F (2009) Effects of receptor-mediated endocytosis and tubular protein composition on volume retention in experimental glomerulonephritis. Am J Physiol Renal Physiol. 296:F902-911

5. Biniossek ML, Niemer M, Maksimchuk K, Mayer B, Fuchs J, Huesgen PF, McCafferty DG, Turk B, Fritz G, Mayer J, Haecker G, Mach L, Schilling O (2016). Identification of protease specificity by combining proteome-derived peptide libraries and quantitative proteomics. Mol Cell Proteomics 15:2515-2524

6. Rawlings ND, Barrett AJ, Finn R. Twenty years of the merops database of proteolytic enzymes, their substrates and inhibitors (2016) Nucleic Acids Res 44:D343-350

7. Gomez-Auli A, Hillebrand LE, Biniossek ML, Peters C, Reinheckel T, Schilling O (2016) Impact of cathepsin b on the interstitial fluid proteome of murine breast cancers. Biochimie 122:88-98

8. Alli AA, Song JZ, Al-Khalili O, Bao HF, Ma HP, Alli AA, Eaton DC. Cathepsin b is secreted apically from xenopus 2f3 cells and cleaves the epithelial sodium channel (enac) to increase its activity (2012) J Biol Chem 287:30073-30083
